# Awareness of diabetes and diabetic retinopathy among a group of diabetic patients in main public hospitals in Damascus, Syria during the Syrian crisis

**DOI:** 10.1186/s12913-019-4375-8

**Published:** 2019-08-05

**Authors:** Ammar Hamzeh, Ghaith Almhanni, Yazen Aljaber, Rana Alhasan, Raneem Alhasan, MHD Imadaldin Alsamman, Nawras Alhalabi, Yousra Haddeh

**Affiliations:** 1 Department of Ophthalmology, Al-Mouwassat University Hospital, Damascus, Syria; 2 Faculty of Medicine, Damascus University, Damascus, Syria; 3 Faculty of Medicine, Syrian Private University, Damascus, Syria

**Keywords:** Awareness, Knowledge, Diabetes mellitus, Diabetic retinopathy, Syria, Syrian crisis

## Abstract

**Background:**

The awareness of diabetes mellitus (DM) and its complications, especially diabetic retinopathy (DR), is the key to reducing their burden. This study aimed to assess both the awareness of diabetic outpatients and their action towards periodic eye exam, and to determine the causes of non-compliance amongst patients who were aware. Because the Syrian Crisis affected all aspects of Syrians’ life, the study aimed to determine the crisis’ effects on patients’ care-seeking behavior. Our study was the first step in paving the way of prevention strategies.

**Methods:**

This observational cross-section study was conducted on 260 patients with DM who were visiting the four main hospitals in the Syrian capital, Damascus between August and November 2017.

**Results:**

The mean (±SD) age of participants was 54.3(±12.8) years. Females were more than half (56.2%). The majority were from areas outside Damascus (72.3%). The mean (±SD) DM duration was 10.6 (±7.1) years. Almost all patients (93.8%) thought that DM could affect the eye. 67.3% believed that it could cause blindness. 86.9% of the patients conceived that DM patients should visit an ophthalmologist regularly. 37% did not visit any ophthalmologists at all, while 63% reported they had visited their ophthalmologists. Only 21.5% had a regular eye exam. Gender, educational level, economic status, province, and family history of DM had statistically an insignificant relation with an ophthalmologist visit. The preponderance of the patients who haven’t visited regularly did not appreciate the necessity of regular eye exam. Diabetic neuropathy was the most common complication of DM that patients were aware of (92%) and suffered from (56.5%). Meanwhile, regarding the effects of the Syrian Crisis: 41.2% of diabetic patients had stopped their medications for at least one month, mainly because the drugs were unavailable (74.7% of them), as some drug companies had been destroyed. Half of the patients had struggled to reach a medical care center. Half of the patients had been displaced, two-third of them were from outside Damascus.

**Conclusion:**

A screening program for DR should be initiated. Also, awareness about DM and its complications, especially DR, should be raised through doctors and media.

**Electronic supplementary material:**

The online version of this article (10.1186/s12913-019-4375-8) contains supplementary material, which is available to authorized users.

## Background

Diabetes mellitus (DM) is a systemic disease characterized by a chronic increase in blood glucose. Recently, it is a global burden due to its systemic complications that affect different parts of the body [1].

The prevalence of DM in the Middle East Region (MER) was estimated to be 10.5% by a recent systematic review of the prevalence of cardiovascular risk factor in the Middle East [2]. DM prevalence in 2015 was estimated to be 415 million among people aged 20–79 years. The developing countries are the most affected, with 75% of the estimated number. By 2040, the predicted number of diabetic patients is 642 million. According to the International Diabetic Federation (IDF) press release in 2017, the Middle East and North Africa Region have approximately 35.4 (24.3–47.4) million people with DM, aged 20–79. Over 40.6% of them are undiagnosed, and 67% live in urban areas. 83.9% of diabetic patients in the region are from low- or middle-income countries [3]. In Syria, one population-based survey in Aleppo city in 2010 estimated that DM was 15.6% based on fasting plasma glucose and 14.8% based on HbA1c [4].

DM has many complications, including cardiovascular disease, neuropathy, nephropathy, and diabetic retinopathy (DR). DR is the main cause of visual impairment in middle-aged and elderly people [5]. Over one-third of estimated diabetic patients have signs of DR, and severe signs of DR are presented in a third of them. Severe signs include non-proliferative diabetic retinopathy (NPDR) or proliferative diabetic retinopathy (PDR) or the presence of diabetic macular edema (DME) [6]. Also, about 75% of patients with more than 20 years of DM are expected to develop DR or DME [7].

Vision problems costs are rising, with an estimated cost of $3 trillion in 2010 to about $3.6 trillion in 2020 [8]. The greatest burden on visual impairment worldwide is related to people aged over 50 years (65%), and this scale composes 82% of blindness [9]. Lack of insurance coverage and unaffordable costs is the main barriers to the best eye healthcare in the developed countries. However, unawareness of the importance of periodic eye examinations is also an important factor [10].

Diabetic patients’ awareness of DM complications, especially DR, has a huge impact on patients’ behavior. Early diagnosis of DR leads to early treatment and to decrease in visual impairment. Regular eye examinations should be sought in diabetic patients for DR screening [11]. Community awareness is the most important factor in the success of any screening program. Diabetic patient’s awareness of DR still not enough in most countries, including developed and developing ones [12].

To the best of our knowledge, there is no previous study of diabetic patients’ awareness of DR and appropriate care-seeking behaviors in Syria. Generally, there is no national program for screening people for DM or its complications. We aim to assess patients’ knowledge and education about DM and its complications, especially DR.

## Methods

We designed a questionnaire (which is attached as Additional files 1 and 2) that included an introduction which presented questions about socio-demographic features including age, gender, educational level, marital status, and socioeconomic status, followed by four sections (Table 1).

**Table 1:** Participant demographics and socioeconomic properties

	Number	Percent
Gender		
Male	114	43.8%
Female	146	56.2%
Educational level		
Illiterate	67	25.8%
Primary school (6–12 years)	82	31.5%
Secondary school (12-15 years)	37	14.2%
High school (15-18 years)	32	12.3%
University (above 18 years)	42	16.2%
Economic status		
Low economic status (do not suffice essential needs)	89	34.2%
Moderate status (suffice essential needs)	116	44.6%
Good (suffice essential needs with some and prosperities)	52	20.0%
Very good	3	1.2%
Marital state		
Married	201	77.3%
Widow	34	13.1%
Single	18	6.9%
Divorced	4	1.5%
Female Work status	146	
White-collar job	24	16.0%
Blue-collar job	11	8.0%
Housekeeper (not worker)	111	76.0%
Male Work Status	114	
Don’t work	12	10.0%
Blue-collar job	60	53.0%
White-collar job	42	37.0%
Lives in		
Damascus	72	27.7%
Outside Damascus	188	72.3%

The first section included several key questions that assess the patients’ knowledge and education about the disease (Tables 2 and 3). In the second section, we assessed the patients’ diabetic status by asking about the duration of DM, age of onset, DM medication type and usage, and how they monitor their diabetic status. In addition, we asked about the presence of any diabetic complications including diabetic neuropathy, kidney disease, previous stroke, previous ischemic heart disease, concomitant blood hypertension or hyperlipidemia, and family history of diabetes (Tables 2, 4). The third section had four short questions, through which we tried to figure out some effects of the Syrian Crisis on patients’ ability to access their medications and reach medical care center (Table 6). The last part concentrated on patients’ DR status and their action toward diabetic eye disease (Table 8).

**Table 2:** Patients’ awareness of diabetic eye disease

Part 1: Awareness to diabetic eye disease	Yes	No	Do not know
	n (%)	n (%)	n (%)
Do you think that Diabetes could affect the eye?	244 (93.8)	4 (1.5)	12 (4.6)
Do you think that Diabetes could cause blindness?	175 (67.3)	9 (3.5)	76 (29.2)
Do you think that diabetic patient should visit an eye doctor periodically?	226 (86.9)	12 (4.6)	22 (8.5)
If yes, do you know the period?			
When vision problems occur	10 (4.4)		
Once a year	175 (77.8)		
Every two years	18 (8.0)		
Don’t know	22 (9.8)		
	225		
Do you think that control of blood glucose is enough to treat diabetic retinopathy without any farther intervention?	158 (60.8)		102 (39.2)
Do you know what are the treatment modalities for diabetic retinopathy?			
Don’t know	148 (56.9)		
Laser, surgery or injections in the eye	111 (42.7)

**Table 3:** Awareness to other diabetic complications

Do you think that diabetes could be a risk for?	Yes	No	Don’t know
	n (%)	n (%)	n (%)
Heart disease	197 (75.8)	21 (8.1)	42 (16.2)
Stroke	125 (48.1)	28 (10.8)	107 (41.2)
Kidney disease	142 (54.6)	46 (17.7)	72 (27.7)
Diabetic neuropathy (Numbness^a^)	240 (92.3)	4 (1.5)	16 (6.2)
Leg stroke^b^	174 (66.9)	15 (5.8)	71 (27.3)

^a^ Numbness was explained to the patients as loss of feeling in hands and feet and abnormal spontaneous sensations (paresthesia)

^b^ (limb ischemia)

**Table 4:** Patients ‘diabetes Status

Part 2: Patients ‘diabetes Status		
Diabetic duration	10.67 ± (7.103) year	
Age of onset	43.70 ± (11.952) year	
	n (%)	n (%)
DM type	Type 1: 20 (7.7%)	Type 2: 239 (92.3%)
How did you know that you have DM?		
Diabetic symptoms like polydipsia and polyurea	164 (63.1%)	
After psychological stress	44 (16.9%)	
Regular checkup	38 (14.6%)	
Incidentally	8 (3.1%)	
After pregnancy	4 (1.7%)	
What type of diabetic medication you take?		
One type of medication	0	125 (52.4%)
Two type medications	0	74 (30.9%)
Insulin injections	19(95%)	29 (12.1%)
Untreated	1(5%)	11 (4.6%)
Are you on diet for diabetes?	Yes, 142 (54.6)	
	No, 118 (45.4)	
How do you assess your diabetic control?	not controlled	90 (34.6)
	moderately controlled	91 (35)
	controlled	79 (30.4)
Based on what did you assess your diabetic control?	glucose measurement and doctor assessment	140 (53.9)
	symptoms’ relief and subjective sense.	116 (44.6)

The questionnaire content was assessed by three internal medicine specialists from the Faculty of Medicine, Damascus University. The questionnaire applicability and validity were assessed after conducting a pilot study on 15 patients; some questions were edited accordingly. Internal Consistency for the pilot study questionnaire was assessed using Chronbach’ s alpha (0.711), which reflected the good result. Ethical approval was obtained from the Damascus University Ethical Committee for Medical Faculties.

Study participants were diabetic patients visiting internal medicine clinics for non-ophthalmic complains. Samples were collected from outpatient departments in the four main public hospitals in Damascus, which were Al-Mouwassat University Hospital, Al Assad University Hospital, Ibn Al-Nafees Hospital, and Red Crescent Hospital. Through 3 months (August 2017 to November 2017), we were able to collect 260 forms. During data collection, investigators administered questionnaire forms after taking the patients’ informed consents and explaining the questions to the patients. In case a patient was illiterate, the investigator filled the form from a patient directly.

Statistical analysis was done using SPSS v23 (IBM Corporation, Armonk, NY, USA). Percentages and numbers were calculated by summarizing categorical and nominal data. Chi-square test was used for finding significant differences in participants’ characteristics. The level of significance was set at *p* < 0.05.

## Results

A total of 260 patients accepted to participate. Numbers of males were 114 (43.8%), and females were 146 (56.2%). The mean age was 54.3 (±12.8) years. The mean number of years since diagnosis of DM was 10.6 (±7.1) years, with a maximum of 40 years and a minimum of less than 1 year. The demographic data showed that 27.7% of the patients lived in Damascus.

The educational level of patients was low: 25.8% were illiterate, 31.5% reached primary school, 14.2% reached middle school, 12.3% reached high school, and 16.2% reached university. 34.2% of patients reported low economic status (do not suffice essential needs), 44.6% reported moderate status (suffice essential needs). Whereas 20.0 and 1.2% reported good and very good economic status, respectively (suffice essential with some and enough prosperity). 52.7% of the patients had or used to have freelance jobs like barber or salesman. Most of the women did not work, and they were housekeepers (76%). Most patients were married 77.3, 13.1% were widowed, 6.9% were single, and 2.7% were divorced. (Table 1) shows the socioeconomic properties of the study’s sample.

### Awareness of diabetic eye disease

Although almost all patients, 93.8%, were aware that DM could affect the eye. However, only 67.3% were aware that it could cause blindness, 29.2% did not know that DM could cause blindness, and 3.5% assured that it would not cause blindness (Table 2). We asked the patients several other questions to assess their knowledge of the disease and how it’s managed: 86.9% of patients thought that diabetic patients should visit an ophthalmologist on a regular basis, 77.8% of them thought that they should do so every one year, 8% every two years, 9.8% did not know, and 4.4% answered “when there is an eye problem”.

More than half of the patients did not know any of the treatment modalities for DR (56.9%) such as laser photocoagulation, surgery, intravitreal injection. While the rest (42.7%) had heard about at least one of these treatment modalities. 60.3% of patient thought that the control of blood glucose is enough to control DR without any further intervention.

We asked patients if they knew other diabetic complications (Table 3). The diabetic neuropathy (numbness) was the most prevalent complication of DM that patients knew well followed by heart disease. The Diabetic neuropathy explained to the patients as loss of feeling in hands and feet (numbness) and abnormal spontaneous sensations (paresthesia).

### Patients ‘diabetes status

The mean period of having DM for affected patients was 10.6 ± 7.1 years, most of them (92%) had type 2 DM. Regarding initial diagnosis, more than half (63.1%) had been diagnosed when they complained from diabetic symptoms like polydipsia and polyuria, 16.9% after psychological stress, 14.6% were diagnosed with a regular checkup at their doctors, 3.1% were diagnosed incidentally and 2.3% after pregnancy. 92% had visited their doctors for symptoms related to DM, 53.8% had hypertension, and 34.2% had hyperlipidemia, In addition, 63.8% reported the presence of DM in another family member. Patient’s thoughts about their DM control were: 34.6% thought that their DM was not controlled, 35% thought that it was moderately controlled and 30.4% thought it was controlled. We asked the patients about their basis in assessing their diabetic control. The answers were: 53.9% depended on glucose measurement and doctor assessment (objective tools), while 44.6% depended on their symptoms’ relief and their subjective sense about the glucose level (subjective tools).

Regarding diabetic medications usage, in patients with type 2 DM, 52.4% were using one type of diabetic medication, 30.9% were using two medications, while 12.1% of type 2 were taking insulin injections injection with or without any other medications, the majority of type 1 DM 19(95%) were on insulin injection with or without any other medication. Untreated patients were 11(4.6%) of type 2 and 1(5%) of type 1 DM. 55% of patients were on a diet for DM (Table 4).

We tried to assess the prevalence of diabetic complications among our patients. The most common prevalent was diabetic neuropathy, 56.5% (147/260) (Table 5).

**Table 5:** Patients ‘diabetes Status

		n (%)
Have you visited a doctor for diabetes before?	yes	241 (92.7)
	no	16 (6.2)
Do you have any of the following diabetic complications?	Diabetic neuropathy (numbness)^a^	147 (56.5)
	Kidney disease	39(15)
	Stroke	13 (5)
	Heart disease	30 (11.5)
	leg stroke ^b^	37 (14.2)
Do you Have:	Hypertension	140 (53.8)
	Hyperlipidemia	89 (34.2)
	Family history of Diabetes	166 (63.8)

^a^ Numbness was explained to the patients as loss of feeling in hands and feet and abnormal spontaneous sensations (paresthesia)

^b^ (limb ischemia or lower limbs arterial occlusion)

Patients’ knowledge about other diabetic complications was more evident in those having these complications. Most of the patients were aware that loss of feeling in the peripheral limbs (numbness) and abnormal spontaneous sensations (paresthesia) are an adverse effect of DM as most of them have this problem.

### The Syrian crisis effects

The effects of the Syrian Crisis on People with diabetes were as follows: 41.2% of diabetic patients had stopped their medications for at least one month once, through the past 7 years, 74.8% of them attributed their stop to the drug unavailability, about half of the patients were forced to change their drug brand names as some drug companies were closed. Half of the patients had struggled to reach a medical care center; 58.1% of the participants were displaced due to the Crisis (Table 6).

**Table 6:** The Syrian Crisis effects on patients

Part 3: The Syrian Crisis effect		
	n (%)	n (%)
Have you stopped your medication for at least one month?	Yes, 107 (41.2)	
	No, 153 (58.8)	
Why have you stopped your medication?	drugs were unavailable	80 (74.8)
	Feeling it is not necessary	16 (15.0)
	high price	11 (10.3)
	Did not stop	153 (58.8)
Have you changed your diabetic medication because it was not available? another brand name	Yes, 142 (54.6)	
	No, 118 (45.4)	
Have you suffered to reach a medical care center?	Yes, 130 (50.0)	
	No, 130 (50.0)	
Have you displaced due crisis?	Yes, 151 (58.1)	
	No, 109 (41.9)	

### Sources of awareness

Patients’ answers about their sources of information regarding DM and DR were other diabetic patients (40%), followed by doctors (32.4%), while (10%) from media like TV or internet and (34.6%) from undermined sources (Table 7).

**Table 7:** Patients’ sources of information about Diabetes Milletus and Diabetic Retinopathy

How did you acquire your information about diabetes?	n (%)
Other diabetic patients	68 (40.0)
Doctors	55 (32.4)
Family and friend	30 (17.6)
Media	17 (10.0)
Total	170
Undetermined	88 (34.6)

### Practice toward the diabetic eye

59.6% of diabetic patients reported having an eye problem due to DM; most of the patients could not determine the level of their DR (72.4%) (Table 8). 36.9% had not visited an ophthalmologist at all, while 63.1% of the patients have had visited an ophthalmologist at least once. When we asked about the regularity of the visit, 65.9% of patients who had visited an ophthalmologist did not visit regularly, and 34.1% regularly visited (Table 9). We tried to discover the reason by asking the patients who did not regularly visit why, 40.5% admitted that they did not appreciate the necessity of the eye examinations, 15.2% for financial reasons, 13.9% reported having difficulties in reaching a medical center and 30.4% for undetermined reasons.

**Table 8:** Practice toward diabetic eye disease

Part 4: Practice toward diabetic eye disease		
	n (%)	n (%)
Do you have an eye problem due to DM?	Yes, 155 (59.6%)	
	No, 105 (40.4%)	
Have you visited an ophthalmologist before	Yes, 164 (63.1%)	
	No, 96 (36.9%)	
If you answered yes to the previous question, Do you visit an ophthalmologist regularly?	Yes, 56 (34.1%)	
	No, 108 (65.9%)	
	Total, 164	
Why you don’t visit an ophthalmologist regularly? (of 108 patients)	do not understand the necessity	32 (40.5%)
	High price	12 (15.2%)
	difficulties in reaching a medical center	11 (13.9%)
	Other^a^	24 (30.4%)
	Total	79
	Missing	29
What is the degree of your diabetic retinopathy?	Mild	6 6.1%
	moderate	16 16.3%
	severe	5 5.1%
	don’t know	71 72.4%
	total	98
	Missing	162

^a^ Other reasons contained special reason to the patients like fear of revealing a hidden disease also a special thought that they believe in it

**Table 9:** Patients’ compliance of visiting and ophthalmologist

	visited an ophthalmologist for different complains	Never visit an ophthalmologist	Total
	n (%)		
Visiting an ophthalmologist regularly			
Yes	56 (34.1%)	N/A	
No	108 (65.9%)	N/A	
	164	96	260

Patients with DR reported that they have other complications included but not limited to numbness -in hands and feet- (diabetic neuropathy) (63%), kidney disease 19.3%, leg stroke (limb ischemia) 18%, heart disease 14.8%, and previous stroke 0.05%.

We compared the patients who visited an ophthalmologist regularly to their answer to the question about their awareness toward visiting regularly (Table 10), Only 24.7% of patients who said that diabetic patients should visit regularly stated that they do visit an ophthalmologist regularly. Despite 75.3% (170/226) were aware that they should visit regularly they did not visit on a regular basis.

**Table 10:** Awareness and patient compliance of visiting to an ophthalmologist

*p* > 0.1	Patients who visit an ophthalmologist regularly			Never visit an ophthalmologist	Total
		YES, I do	NO, I don’t		
The awareness regarding regular visit to an ophthalmologist.	YES, they should	56 (24.7%)	102	68	226 (86.9%)
	NO, don’t know	0	6	28	34
		56 (21.5%)	108	96 (37%)	260

Patients’ regular visit to an ophthalmologist was not statistically associated regarding patients’ gender (Chi-square = 2.598, *p* = 0.206), educational level (Chi-square = 9.233, *p* = 0.323), economic status (Chi-square = 9.341, *p* = 0.155), province (Chi-square = 0.716, *P* = 0.699) or family history of DM (Chi-square = 0.72, *p* = 0.965).

In addition, using also Chi-square test of significance, regarding the awareness status that states “DM could affect the eye”, was not significantly associated with patients’ gender (Chi-square = 1.616, *p* = 0.446), economic Level (Chi-square = 2.040, *p* = 0.916), DM type (Chi-square = 0.344, *p* = 0.842), patients’ educational level (Chi-square = 10.615, *p* = 0.225). In addition, Chi-square test of significance showed the awareness of DR could lead to blindness was only significantly associated patients’ educational level (Chi-square = 16.705, *p* = 0.033) while were not significantly associated with gender, economic level, and DM type.

## Discussion

The demographic data showed that most of the patients lived in areas other than Damascus. This reflected the substantial damage that affected most of the country due to ongoing war and loss of health care services in conflict areas. Most of the patients had low socioeconomic status, as public hospitals provide free or low-priced services, in addition, the educational level of patients was also low and a quarter of them were illiterate, and most of them had freelance jobs which reflect them being in working age.

The sample of our study reflected the awareness of diabetic patients who were visiting hospitals due to different complaints. Thus, we cannot generalize our results to all diabetic patients who did not suffer from any complaints and did not visit any clinics.

Regarding DM diagnosis, only 14.6% were diagnosed by regular checkups at their doctors, while the rest have had symptoms that motivated them to undergo a fasting glucose test. This may increase the period between the disease commence and the diagnosis, which led to more ominous diabetic complications. In the 2015 clinical practice guidelines of the American Association of Clinical Endocrinologists and the American College of Endocrinology, it is recommended that screening for DM should start at ≥45 years without other risk factor or earlier in the presence of other risks such as high BMI or hypertension [13].

### Awareness and usual practice of patients toward diabetic eye

In our study, almost all patients (93.8%) thought that DM could affect the eye, in comparison, in Saudi Arabia 75.62% of those with DM were aware that DM could cause eye disorders and the sample was collected from hospitals similar to our study [14], while awareness of diabetic retinopathy in Turkey was 88.1% of diabetic patients who were visiting outpatient clinics [15]. Awareness that DR is a diabetic complication of the eye and can result in blindness among urban diabetic patients’ population in China was 36.6% [16]. The awareness varied from a population to another due to cultural differences. Also, In Bangladesh, 63% of type 2 diabetic patients who were visiting tertiary care hospital were aware that DM could cause blindness [17] which is similar to our study result (67.3%).

It was crystal clear that awareness increased when diabetic patients were visiting hospitals. On the other hand, diabetic patients who were visiting hospital had complaints mostly attributed to systemic diabetic complications. In our study, almost all patients thought that DM could affect the eye. This may be attributed to the fact that all our patients were visiting hospitals, and they may have heard about the possibility of eye harm due to DM.

Despite the high awareness (86.9%) of the patients who thought that diabetic patients should visit ophthalmologists regularly, only 24.7% had a regular eye exam. While in the same previously mentioned study at tertiary care hospital in Bangladesh, only 49% were aware of the need of periodic eye screening exam for diabetic eye complication and only 37% reported that they had performed it annually [17]. A lot of excuses hindered patients from visiting an ophthalmologist; the major one was the lack of knowledge; besides, it came the chronicity nature of DR and the long latent period to symptoms’ manifestations.

(96/260) 37% of our study participants had never visited an ophthalmologist, in another study at a hospital in Ghana, 34.5% had not visited ophthalmology since diagnosis of their DM [18]. Of patients who said that diabetic patients should visit regularly, only 24.7% stated that they visited an ophthalmologist regularly, while 75.3% did not, this gap between awareness and practice could be attributed to several elements. We tried to discover this gap between awareness of regular visit and an actual regular visit by asking the 108 patients who did not regularly visit, why they did not. Most answers were because they did not appreciate the necessity of the eye examination (40.5%). The lack of patients’ understanding could be attributed to the low communication between doctors and their patients or low educational status of patients, which increases the barriers between doctors and their patients. There was no statistical difference between educational level compared with their practice toward a periodic visit to an ophthalmologist (Chi-square test: *p* > 0.1) in our sample; this could be partially attributed to a low number of patients who had reached university level.

### The effects of the Syrian crisis and diabetic patients

Most patients in Syria had suffered from periods when some medications were not available due to damage to some drug companies, especially in Aleppo city in Syria. In our study, 41.2% of diabetic patients had stopped their medications for at least one month, and 74.8% of them attributed their stop to the drug unavailability.

While 68.6% of the patient came from areas other than Damascus, 58.1% of all patients were displaced because of the crisis (85.4% from outside Damascus and 14.6% from Damascus), of those displaced, 68.8% had difficulties in reaching health care providers (Tables 11 and 12).

**Table 11:** The effects of the Syrian Crisis and Diabetic Patients

*p* < 0.05	Have you displaced due crisis?		Total
	yes	no	
	n (%)	n (%)	
Province			
Damascus	22 (14.6)	50	72
Outside Damascus	129 (85.4)	59	188
Total	151 (58.1)	109 (41.9)	260

**Table 12:** The effects of the Syrian Crisis and Diabetic Patients

*p* < 0.05	Have you displaced due crisis?		Total
	yes	no	
	n (%)	n (%)	
Have you suffered to reach a medical care center?			
Yes	104 (68.8)	26	130
No	47 (31.1)	83	130
Total	151	109	260

### Patients source of knowledge

The most common sources of information were other diabetic patients (40%), followed by doctors (32.4%) and the media with only 10%. Compared to the Bangladesh study, the two most common sources were friends and neighbors (51%), family (49%), followed by electronic media (46%) [19]. Media is a good source of information about diseases, and it should be invested more in spreading health awareness and diseases prevention.

## Conclusion

Preventive plans should be designed to spread knowledge among diabetic patients and the general population. Doctors may refer patients for screening for diabetic retinopathy, but they should have better communication with patients, in addition to a thorough explanation about diabetes and its complications, especially diabetic retinopathy. As most patients reported the low contribution of media as a source of their knowledge, mass media like TV and radio and social media should broadcast programs that increase awareness of people about diabetes and raise their knowledge about diabetic complications.

## Additional files

Additional file 1: Questionnaire: we designed this questionnaire for our study. It included an introduction which presented questions about socio-demographic features. There were four sections in the questionnaire .The first section included several key questions that assess the patients’ knowledge and education about the disease. In the second section, we assessed the patients’ diabetic status by asking about the duration of DM, age of onset, DM medication type and usage, and how they monitor their diabetic status. In addition, we asked about the presence of any diabetic complications including diabetic neuropathy, kidney disease, previous stroke, previous ischemic heart disease, concomitant blood hypertension or hyperlipidemia, and family history of diabetes. The third section had four short questions, through which we tried to figure out some effects of the Syrian Crisis on patients’ ability to access their medications and reach medical care center. The last part concentrated on patients’ DR status and their action toward diabetic eye disease. (DOCX 19 kb)

Additional file 2: The manuscript data: it contains raw data of our results. (XLSX 51 kb)

## Data Availability

The datasets used and/or analyzed during this study are available from the corresponding author on any request. Also it was attached as additional file.

## Abbreviations

DM: Diabetes mellitus
DME: Diabetic macular edema
DR: Diabetic retinopathy
HbA1c: Hemoglobin A1C
IDF: International Diabetic Federation
MER: Middle East Region
NPDR: Non-proliferative diabetic retinopathy
PDR: Proliferative diabetic retinopathy
TV: Television
