# Supplementary material for: Awareness of diabetes and diabetic retinopathy among a group of diabetic patients in main public hospitals in Damascus, Syria during the Syrian crisis
Source: BMC Health Serv Res. 2019 Aug 5;19:549. doi: 10.1186/s12913-019-4375-8 (PMC6683401; doi:10.1186/s12913-019-4375-8)
Supplement: Supplementary file 1 — Questionnaire: we designed this questionnaire for our study. It included an introduction which presented questions about socio-demographic features. There were four sections in the questionnaire .The first section included several key questions that assess the patients’ knowledge and education about the disease. In the second section, we assessed the patients’ diabetic status by asking about the duration of DM, age of onset, DM medication type and usage, and how they monitor their diabetic status. In addition, we asked about the presence of any diabetic complications including diabetic neuropathy, kidney disease, previous stroke, previous ischemic heart disease, concomitant blood hypertension or hyperlipidemia, and family history of diabetes. The third section had four short questions, through which we tried to figure out some effects of the Syrian Crisis on patients’ ability to access their medications and reach medical care center. The last part concentrated on patients’ DR status and their action toward diabetic eye disease. (DOCX 19 kb) [file 12913_2019_4375_MOESM1_ESM.docx]

Socioeconomic properties

1. Gender

- male
- female

1. Educational level

- illiterate
- primary school(6-12years)
- Secondary school(12-15years)
- high school(15-18years) 32
- University (above 18 years)

1. Economic status

- low economic status (do not suffice essential needs)
- moderate status (suffice essential needs)
- good (suffice essential needs with some and prosperities)
- very good

1. Marital state

- married
- widow
- single
- divorced

1. Work status

- White-collar job
- Blue-collar job
- don’t work (Housekeeper for women)

1. Lives in

- Damascus
- Outside Damascus

**PART 1 Awareness of diabetic eye disease**.

1. Do you think that Diabetes could affect the eye? Yes no Don’t know
2. Do you think that Diabetes could cause blindness? Yes no Don’t know
3. Do you think that diabetic patient should visit an eye doctor periodically? Yes no Don’t know
4. If yes, do you know the period?

- When vision problems occur
- once a year
- Every two years
- Don’t know

1. Do you think that control of blood glucose is enough to treat diabetic retinopathy without any farther intervention? Yes, Don’t know
2. Do you know what are the treatment modalities for diabetic retinopathy?

- Don’t know
- Laser, surgery or injections in the eye.

| Do you think that diabetes could be a risk for? | yes | no | Don’t know |
| --- | --- | --- | --- |
| 1. heart disease | yes | no | Don’t know |
| 1. stroke | yes | no | Don’t know |
| 1. kidney disease | yes | no | Don’t know |
| 1. Diabetic neuropathy (Numbness*) | yes | no | Don’t know |
| 1. leg stroke(limb ischemia) | yes | no | Don’t know |

*loss of feeling in hands and feet and abnormal spontaneous sensations (paresthesia)

1. How did you acquired your information about diabetes?,

- other diabetic patients
- Doctors
- Family and friend
- media
- total
- undetermined

**Part 2: Patients ‘diabetes Status**

1. Diabetic duration ……..
2. Age of onset ……..
3. DM type

- Type 1
- Type 2

1. how did you know that you have DM?

- diabetic symptoms like polydipsia and polyurea
- after psychological stress
- regular checkup
- incidentally
- after pregnancy

1. What type of diabetic medication you take?

- one type of medication
- Two type medications
- take insulin injections
- untreated

1. Are you on diet for diabetes?

- Yes
- No

1. how do you assess your diabetic control?

- not controlled
- moderately controlled
- controlled

1. Based on what did you assess your diabetic control?
   - glucose measurement and doctor assessment
   - symptoms’ relief and internal feeling.
2. Have you visited a doctor for diabetes before?

- yes
- no

Do you have any of the following diabetic complications?

1. Diabetic neuropathy(numbness)* YES NO
2. Kidney disease YES NO
3. Stroke YES NO
4. Heart disease YES NO
5. leg stroke (limb ischemia) YES NO
6. Do you Have Hypertension YES NO
7. Do you Have Hyperlipidemia YES NO
8. Do you Have Family history of Diabetes YES NO

**Part 3: The Syrian Crisis effect**

1. Have you stopped your medication for at least one month?

- yes
- no

1. why have you stopped your medication?

- drugs were unavailable
- Feeling it is not necessary
- high price
- Did not stop

1. Have you changed your diabetic medication because it was not available? another brand name

- yes
- no

1. have you suffered to reach a medical care center?

- yes
- no

1. Have you displaced due crisis?

- yes
- no

**Part4: Practice toward diabetic eye disease**

1. do you have an eye problem due to DM?

- yes
- no

1. Have you visited an ophthalmologist before?

- yes
- no

1. If you answered yes to the previous Q n42, Do you visit an ophthalmologist regularly?

- yes
- no

1. If you answered no to the previous Q n43 why you don't visit an ophthalmologist regularly?
   - - do not understand the necessity
     - High price
     - difficulties in reaching a medical center
     - Other PLEASE WRITE IT DOWN…………………………………..
2. What is the degree of your diabetic retinopathy

- Mild
- moderate
- severe
- don't know
